# Interpretation of mitochondrial diversity in terms of taxonomy: a case study of *Hyponephele lycaon* species complex in Israel (Lepidoptera, Nymphalidae, Satyrinae)

**DOI:** 10.3897/zookeys.538.6689

**Published:** 2015-11-19

**Authors:** Vladimir A. Lukhtanov, Asya V. Novikova

**Affiliations:** 1 Department of Karyosystematics, Zoological Institute of the Russian Academy of Sciences, Universitetskaya nab. 1, 199034 St. Petersburg, Russia; 2 Department of Entomology, Faculty of Biology, St Petersburg State University, Universitetskaya nab. 7/9, 199034 St. Petersburg, Russia; 3 Department of Ecology, Evolution and Behavior, the Hebrew University of Jerusalem, Givat Ram, Berman bldg, 91904 Jerusalem, Israel

**Keywords:** adaptation to high/low elevation, biodiversity, *COI*, cryptic species, DNA barcoding, disruptive selection, habitat-related selection, molecular markers

## Abstract

It is difficult to interpret mitochondrial diversity in terms of taxonomy even in cases in which a concordance exists between mitochondrial, ecological and morphological markers. Here we demonstrate this difficulty through a study of Israeli *Hyponephele* butterflies. We show that samples commonly identified as *Hyponephele lycaon* are represented on Mount Hermon in Israel by two sympatric groups of individuals distinct both in mitochondrial DNA-barcodes (uncorrected *p*-distance = 3.5%) and hindwing underside pattern. These two groups were collected in different biotopes. They also tended to be different in length of brachia in male genitalia, although the latter character is variable. We reject the hypothesis that the discovered *COI* haplogroups are selectively neutral intraspecific characters. We hypothesize that they represent: either (1) two different biological species, or (2) a consequence of a strong positive selection acting at intraspecific level and resulting in two intraspecific clusters adapted to low and to high elevations. If we accept the first hypothesis, then provisionally these two haplogroups can be attributed to transpalearctic *Hyponephele lycaon* sensu stricto and to *Hyponephele lycaonoides*, previously known from Iran and East Turkey.

## Introduction

*Hyponephele* Muschamp, 1915 is a large and taxonomically diverse genus of satyrine butterflies. The genus contains 39 species (Eckweiler and Bozano 2011) distributed throughout the Palearctic region, with the highest species diversity found in Central Asia, Iran and Turkey. This group was taxonomically revised by Samodurov with co-authors (Samodurov et al. 1995, 1996, 1997, 1999a, b, 2000, 2001) and by Eckweiler and Bozano (2011).

Within the genus, *Hyponephele lycaon* (Rottenburg, [1775]) is the best known and the most common species broadly distributed in the temperate zone of the Palearctic from Portugal in the west to Far East Russia in the east (Samodurov et al. 2001, Eckweiler and Bozano 2011). In south Palearctic it is replaced by closely related allopatric taxa *Hyponephele maroccana* Blachier, 1908 (North Africa), *Hyponephele galtscha* (Grum-Grshimailo, 1893) (Tajikistan) and *Hyponephele sifanica* (Grum-Grshimailo, 1891) (China) (Eckweiler and Bozano 2011). One more species, *Hyponephele lycaonoides* D. Weiss, 1978 was described from Zagros Mountains in Iran. *Hyponephele lycaonoides* was shown to be sympatric with *Hyponephele lycaon* in Iran (Weiss 1978, Eckweiler and Bozano 2011, Tshikolovets et al. 2014). *Hyponephele lycaonoides* was also reported for Turkey (Koçak 1989, Eckweiler and Bozano 2011), but the reports for Turkey were questioned in the comprehensive analysis of Turkish butterfly fauna made by Hesselbarth et al. (1995). Male genitalia structures are commonly used for distinguishing between *Hyponephele lycaon* and *Hyponephele lycaonoides*, and specimens with long brachia are attributed to *Hyponephele lycaon*, whereas specimens with short brachia are attributed to *Hyponephele lycaonoides* (Weiss, 1978). However, male genitalia are variable in both *Hyponephele lycaon* and *Hyponephele lycaonoides*, and intermediate forms are reported to be common (Eckweiler and Bozano 2011). Moreover, Hesselbarth et al. (1995) considered these traits (the long and short brachia) as intraspecific variations, rather than species-specific characters. Unfortunately, until now nobody used molecular markers to test the non-conspecifity of *Hyponephele lycaon* and *Hyponephele lycaonoides*.

In our study we analysed mitochondrial DNA barcodes and morphological and ecological markers to show that butterflies commonly identified as *Hyponephele lycaon* are represented in Israel by two sympatric groups of individuals. We further discuss different possible evolutionary and taxonomic interpretations of the pattern discovered.

## Materials and methods

In the course of our DNA barcode survey of Israeli butterflies (2012–2015) we found butterflies similar to *Hyponephele lycaon* on Mount Hermon in northern Israel. They were collected in a small area situated between 33°17'12"N, 35°45'49"E, at 1440 m and 33°18'38"N, 35°47'07"E, at 2050 m. The distance between these extreme points of the collecting was 3460 m (measured using Google Earth map). Some of the butterflies were collected in the forest zone at 1450–1600 m above sea level, other were collected in the subalpine zone with predominance of xerophytous vegetation at 1800–2050 m above sea level (Table 1).

**Table 1.** List of *Hyponephele* samples sequenced in the present study.

Haplogroup or taxon	Country	Ecological zone	Pattern of the wing underside	BOLD Process ID	Field ID	GenBank accession #
I	Israel	forest	contrasting	BPAL2756-15	CCDB-17969_A01	KT864697
I	Israel	forest	contrasting	BPAL2757-15	CCDB-17969_A02	KT864698
I	Israel	forest	contrasting	BPAL2758-15	CCDB-17969_A03	KT864699
I	Israel	forest	contrasting	BPAL2760-15	CCDB-17969_A05	KT864700
I	Israel	forest	contrasting	BPAL2761-15	CCDB-17969_A06	KT864701
I	Israel	forest	contrasting	BPAL2765-15	CCDB-17969_A10	KT864702
II	Israel	forest	pale	BPAL2695-14	CCDB-17968_C11	KT864691
II	Israel	subalpine	pale	BPAL2705-14	CCDB-17968_D09	KT864692
II	Israel	subalpine	pale	BPAL2706-14	CCDB-17968_D10	KT864693
II	Israel	subalpine	pale	BPAL2733-14	CCDB-17968_G01	KT864690
II	Israel	subalpine	pale	BPAL2762-15	CCDB-17969_A07	KT864694
II	Israel	subalpine	pale	BPAL2763-15	CCDB-17969_A08	KT864695
II	Israel	subalpine	pale	BPAL2764-15	CCDB-17969_A09	KT864696
*Hyponephele lupinus*	Israel	n/a	n/a	BPAL2719-14	CCDB-17968_E11	KT864688
*Hyponephele lupinus*	Israel	n/a	n/a	BPAL2683-14	CCDB-17968_B11	KT864689
*Hyponephele maroccana*	Morocco	n/a	n/a	BPAL1378-12	CCDB-03030_D12	KT864703
*Hyponephele maroccana*	Morocco	n/a	n/a	BPAL1377-12	CCDB-03030_D11	KT864704
*Hyponephele maroccana*	Morocco	n/a	n/a	BPAL1376-12	CCDB-03030_D10	KT864705

DNA barcodes, 658 bp fragments within mitochondrial gene, *cytochrome oxidase subunit I*
(COI), were sequenced at the Canadian Centre for DNA Barcoding (CCDB, Biodiversity Institute of Ontario, University of Guelph) using standard high-throughput protocol described in deWaard et al. (2008). DNA was extracted from a single leg removed from each voucher specimen employing a standard DNA barcode glass fibre protocol (Ivanova et al. 2006). All polymerase chain reactions (PCR) and DNA sequencing were carried out following standard DNA barcoding procedures for Lepidoptera as described previously (Hajibabaei et al. 2005). Photographs of specimens used in the analysis are available in the Barcode of Life Data System (BOLD) at http://www.barcodinglife.org/. All voucher specimens are deposited at the Zoological Institute of the Russian Academy of Sciences and could be identified by the corresponding unique BOLD Process IDs, that were automatically generated by BOLD, and by GenBank accession numbers (Table 1).

The procedure of phylogenetic inference was described previously (Vershinina and Lukhtanov 2010, Talavera et al. 2013, Lukhtanov et al. 2014, 2015a, b). Briefly, the sequences were aligned using BioEdit version 7.1.7 software (Hall 1999) and edited manually. Phylogenetic relationships were inferred using Bayesian Inference and the program MrBayes 3.2.2 (Ronquist et al. 2012). A GTR substitution model with gamma distributed rate variation across sites and with proportion of invariable sites was specified before running the program as suggested by jModelTest (Posada 2008). Two runs of 10,000,000 generations with four chains (one cold and three heated) were performed. Chains were sampled every 10,000 generations, and burn-in was determined based on inspection of log likelihood over time plots using TRACER, version 1.4 (available at http://beast.bio.ed.ac.uk/Tracer). For comparison we used additional *COI* barcodes of *Hyponephele* downloaded from GenBank (Lukhtanov et al. 2009, Dinca et al. 2011).

Butterfly photographs were taken with Nikon D810 digital camera equipped with a Nikon AF-S Micro Nikkor 105 mm lens. Genitalia photographs were taken with Leica M205C binocular microscope equipped with Leica DFC495 digital camera, and processed using the Leica Application Suite, version 4.5.0 software.

## Results

During a 2012-2015 survey of Israeli fauna, *Hyponephele lycaon*-similar butterflies were found only on Mount Hermon in northern Israel. We never observed *Hyponephele lycaon*-similar butterflies in other parts of Israel, although the distantly related *Hyponephele lupinus* (Costa, 1836) was found not only in the northern, but also in central Israel. Thus, our observations support the finding that the geographic range of *Hyponephele lycaon* species complex is restricted in Israel to the northernmost part of the country (Benyamini 2002).

**Figure 1.**
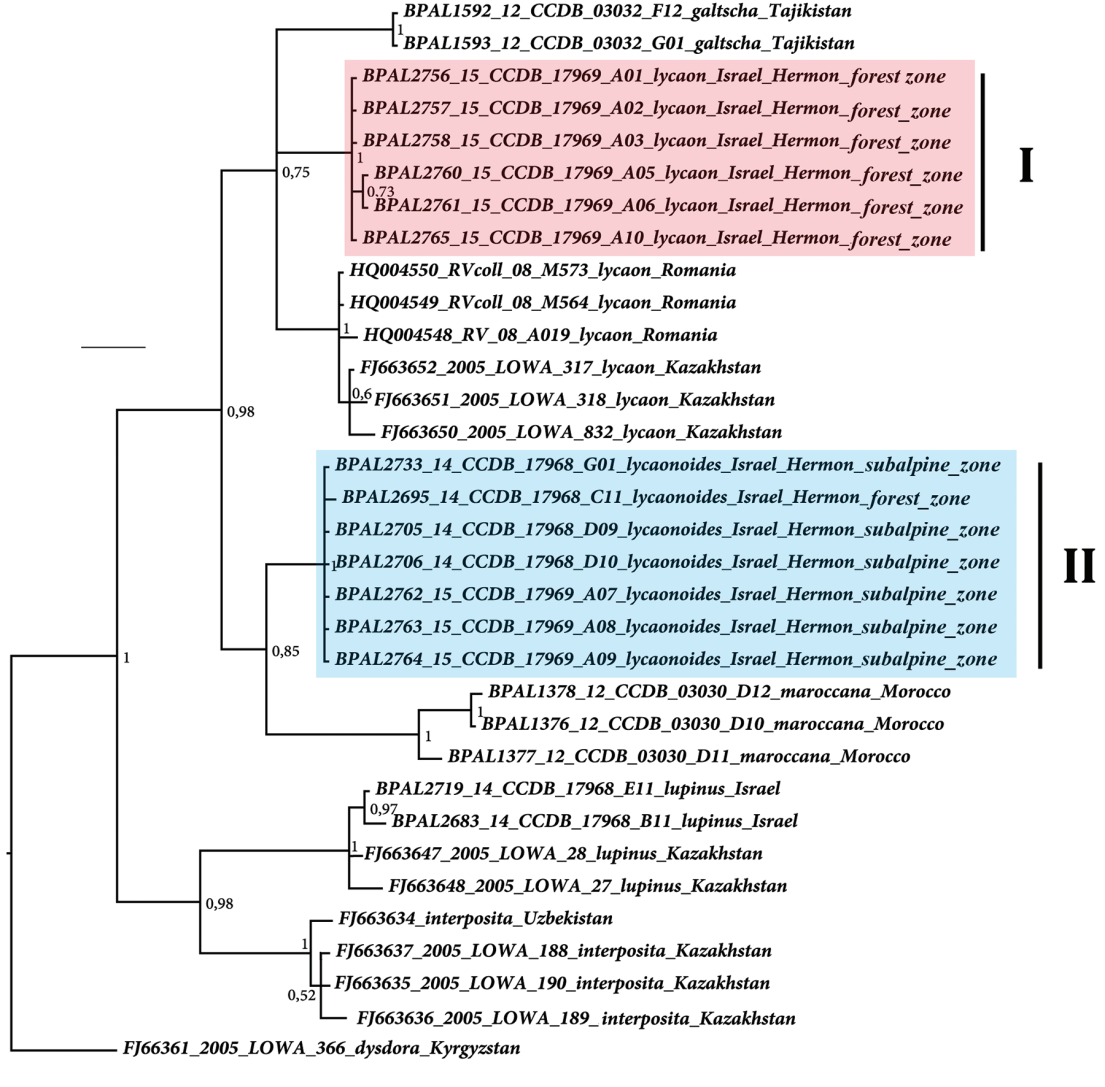
The Bayesian tree of the *Hyponephele lycaon* species complex based on analysis of *COI* DNA barcodes. Numbers at nodes indicate Bayesian posterior probability values. Sympatric haplogroups I and II from Israel are highlighted. Scale bar = 0.2 substitutions per position.

Molecular analysis of *Hyponephele lycaon*-similar samples (Table 1, Fig. 1) revealed two distinct mitochondrial haplogroups (I and II) that were strongly differentiated with respect to the *COI* gene. These two haplogroups differed from one another by 23 fixed nucleotide substitutions in the studied 658 bp fragment of the mitochondrial *COI* gene. When looking at the level of primary polypeptide structure, these differences translate to two fixed amino acid substitutions in the studied fragment. The minimal uncorrected *COI* *p*-distance between these two haplogroups was found to be as high as 3.5 %. *Hyponephele lupinus* from Israel was found to be closely related to *Hyponephele interposita* and distant from all the taxa of the *Hyponephele lycaon* complex.

**Figure 2.**
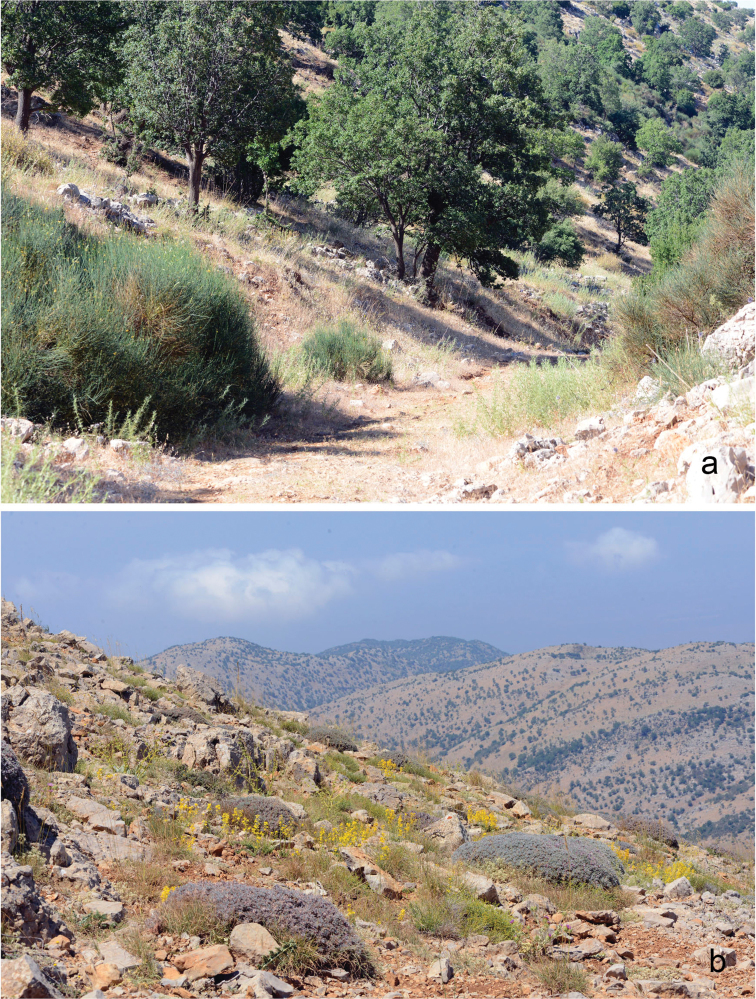
Biotopes on Mount Hermon, Israel where *COI* haplogroups I (**a**) and II (**b**) were collected.

With a single exception (female sample BPAL2695-14|CCDB-17968_C11, Fig. 1, Table 1), the representatives of these two *COI* haplogroups were collected in different biotopes (Fig. 2). The butterflies of haplogroup I were found on grassy slopes in the forest zone (1450-1600 m above see level) (Fig. 2a). The butterflies of haplogroup II were found in steppe lands of the subalpine zone (1800–2050 m alt.), where xerophytous thorny cushion vegetation formed by *Onobrychis cornuta* and *Astragalus* species (Fabaceae) was predominant (Fig. 2b).

Standard χ2-test was used to distinguish between random *vs.* non-random distribution haplogroups I and II in the low (forest) and high (subalpine) zones. Empirical and expected frequencies of *COI* haplogroups I and II in low and high altitude belts were compared (Table 2). The calculated χ^2^ was larger than the tabular value (9.558 *vs.* 6.635, df = 1, 0.01 level of significance). Therefore, we reject the *H_0_* hypothesis and conclude that haplogroup I butterflies are significantly more frequent in the lower zone, whereas haplogroup II butterflies are significantly more frequent in the higher zone.

**Table 2.** Primary data (number of samples) for χ2-analysis of random *vs.* non-random distribution of the *COI* I and II haplogroups in the low (forest) and high (subalpine) zones.

	empirical values		expected values (in case of random distribution)	
	low altitude	high altitude	low altitude	high altitude
*COI* haplogroup I	6	0	3.234	2.772
*COI* haplogroup II	1	6	3.766	3.228

**Figure 3.**
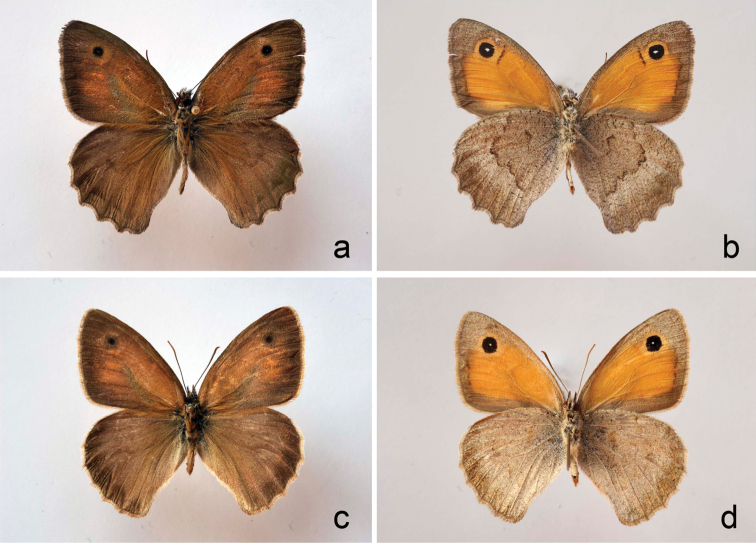
Wing pattern in haplogroup I and II samples from Mt Hermon, Israel. The pictures were taken using diffused daylight **a** sample CCDB-17969_A02, upperside **b** sample CCDB-17969_A02, underside **c** sample CCDB-17969_A09, upperside **d** sample CCDB-17969_A09, underside.

**Figure 4.**
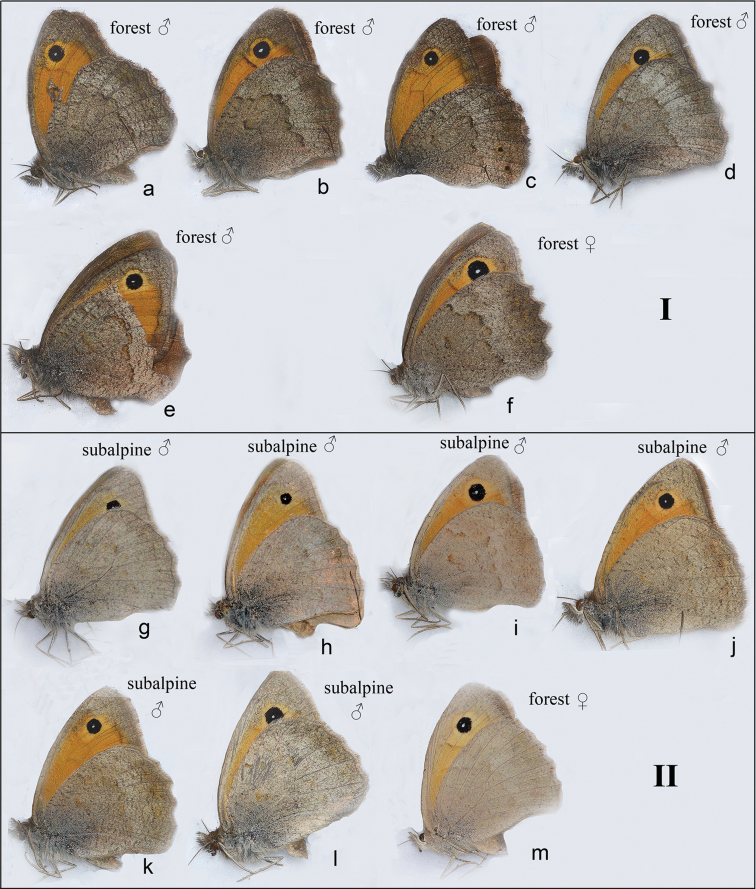
Pattern of the wing underside in haplogroups I and II samples. The pictures were taken using a flash **a** CCDB-17969_A01 **b** CCDB-17969_A02 **c** CCDB-17969_A03 **d** CCDB-17969_A06 **e** CCDB-17969_A10 **f** CCDB-17969_A05 **g** CCDB-17968_C11 **h** CCDB-17968_D09 **i** CCDB-17968_G01 **j** CCDB-17969_A07 **k** CCDB-17969_A08 **l** CCDB-17969_A09 **m** CCDB-17968_C11.

The representatives of these two clusters were also different in the pattern on the hindwing underside (Figs 3 and 4). In haplogroup I this pattern had more contrast with clearly visible medial band, whereas in haplogroup II the hindwing underside was paler and had less contrast.

A standard χ2-test was used to distinguish between random *vs.* non-random association between haplogroups I and II and hindwing underside pattern (Table 3). The calculated χ^2^ of 12.860 was larger than the tabular value (12.860 *vs.* 10.83, df = 1, 0.001 level of significance). Therefore, we reject the *H_0_* hypothesis and conclude that *COI* haplogroup I is significantly associated with contrast pattern of the hindwing underside, whereas *COI* haplogroup II is significantly associated with pale pattern of the hindwing underside.

**Table 3.** Primary data (number of samples) for χ2-analysis of random *vs.* non-random association between the haplogroup I and II and the hindwing underside pattern.

	empirical values		expected values (in case of random distribution)	
	contrast pattern	pale	contrast pattern	pale
*COI* haplogroup I	6	0	2.772	3.234
*COI* haplogroup II	0	7	3.228	3.766

**Figure 5.**
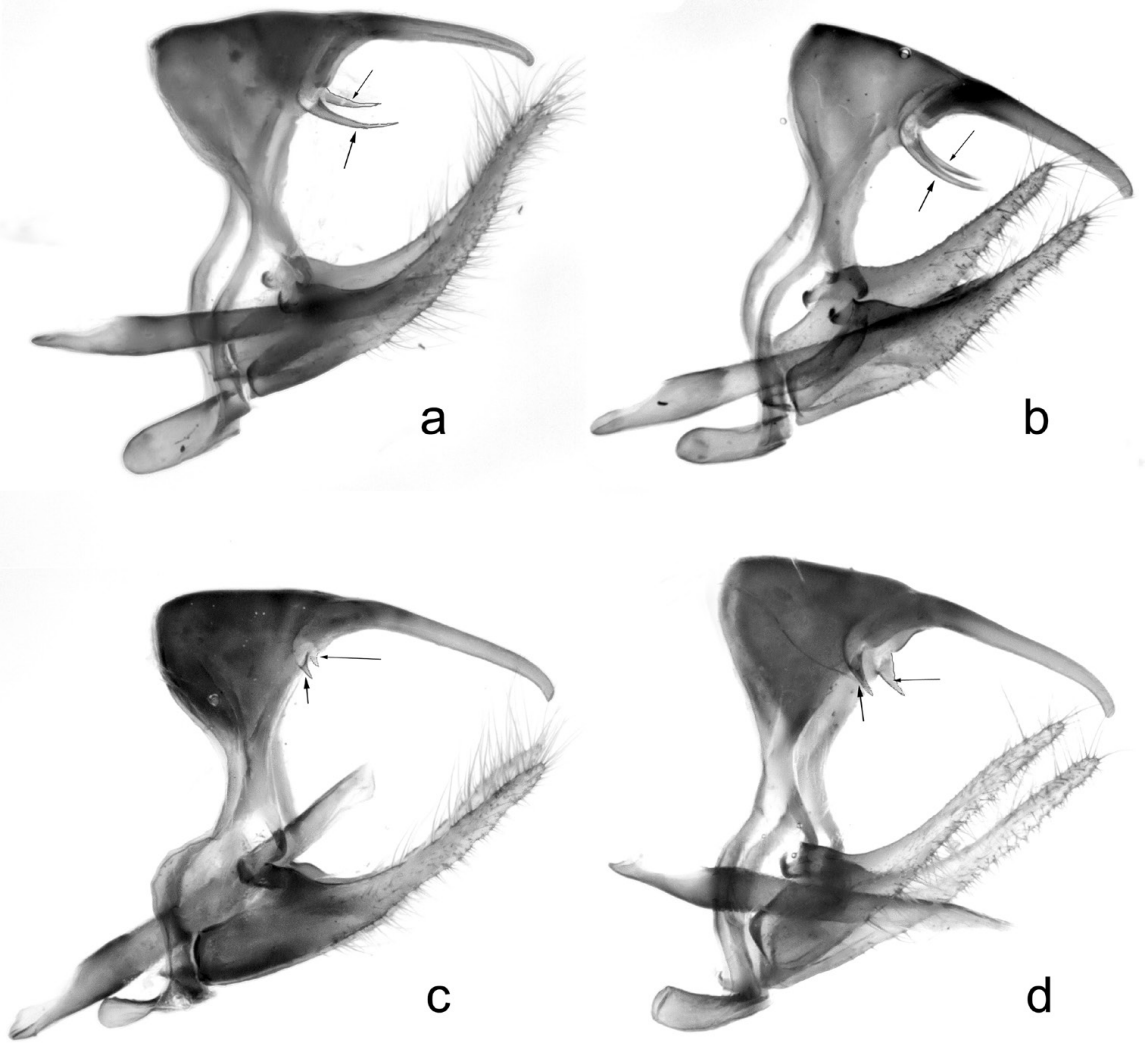
Typical male genitalia in haplogroups I (**a**, **b**) and II (**c**, **d**) from Mt Hermon, Israel. Lateral view. Brachia are indicated by arrow **a** specimen CCDB-17969_A10 **b** specimen CCDB-17969_A01 **c** specimen CCDB-17968_D10 **d** specimen CCDB-17968_D09.

The representatives of these two *COI* haplogroups also tended to be different in the length of the brachia in male genitalia (Fig. 5), although the latter character had high variability. Males of haplogroup I often had long brachia (Fig. 5a, b), whereas males of haplogroup II were mainly characterized by reduced brachia (Fig. 5c, d).

## Discussion

### Evolutionary interpretation of the discovered pattern

The *COI* genetic distance between haplogroups I and II (3.5 %) is higher than the ‘standard’ 2.7–3.0% DNA-barcoding threshold commonly used as a tentative indicator for species distinctness of the taxa compared (Lambert et al. 2005, Lukhtanov et al. 2015a). It is known that *COI* barcodes alone are not sufficient for making any taxonomic decisions, since trees inferred from single markers sometimes display relationships that reflect the evolutionary histories of individual genes rather than the species being studied (Nichols 2001). Mitochondrial introgression (Zakharov et al. 2009) and *Wolbachia* infection (Ritter et al. 2013) can lead to additional bias when inferring taxonomic conclusions based on mitochondrial genes. Typically, multiple molecular markers or a combination of morphological and molecular markers are required for inferring taxonomic hypotheses. In our research such additional information is represented by ecological characteristics (altitude belts). Less attention was attributed to the wing pattern, since we were not sure that it was an independent character. As the wing pattern strongly correlated with the ecology (low versus high elevation), one could hypothesize that the morphological difference is a consequence of phenotypic plasticity, i.e. ability of the same genotype to result in different phenotypes in response to changes in the environment (Price et al. 2003).

Three alternative explanations can account for bimodal sympatric distribution of mitochondrial markers. First, the diverged *COI* sequences may be selectively neutral intraspecific characters. Both preservation of a variety of ancestral haplotypes and mitochondrial introgression due to complex phylogeographic history could be responsible for such a neutral polymorphism (Avise 2000). Second, bimodal sympatric distribution of mitochondrial markers may be a result of a strong positive habitat-related selection working at intraspecific level and resulting in two *COI* clusters associated with different altitude belts (Cheviron and Brumfield 2009). Third, bearers of two diverged haplogroups may represent two different biological species (Avise 2000).

In our case the first hypothesis (neutral polymorphism) can be easily rejected. It predicts that the *COI* haplogroups I and II should be stochastically (i.e. randomly) distributed within high and low altitude belts. This prediction is not supported by χ2-test that demonstrated significantly non-random distribution of the *COI* haplogroups.

The second hypothesis (strong intraspecific positive selection) offers a more exotic, but not improbable, explanation. As *COI* sequence can be translated into a subunit of cytochrome c oxidase, a functional protein in mitochondria involved in energy metabolism (Kirk and Freedland 2011), this gene should be under natural selection (Castoe et al. 2008). Different haplotypes at this locus (or other linked mitochondrial genes) may be favoured in different environments. This could trigger a rapid sweep to fixation of a novel haplotype. This may result in sympatric clusters that differ in mitochondrial genes while exchanging alleles freely throughout the rest of the genome. Interestingly, such groups maintained by habitat-related selection could be considered species according to the genotypic cluster species concept (Coyne and Orr 2004, p. 448–449). The positive habitat-related selection of mitochondrial genome, despite its theoretical plausibility, has so far relatively low empirical support, although there are some data confirming mitochondrial evolution along temperature and altitude gradients (Cheviron and Brumfield 2009, Quintela et al. 2014).

The third hypothesis (two different species) seems to be a more likely explanation in the case of haplogroups I and II, especially if one takes into account the high level of genetic divergences between the haplogroups and concordance between molecular (Fig. 1), ecological (Fig. 2, Table 2) and morphological (Figs 3 and 4, Table 3) characters. More samples, especially from the intermediate elevation (1600–1800 m), and analysis of additional nuclear molecular markers across altitudinal transect will be required in future research to support or to reject the second (positive selection) and the third (two species) hypotheses and to reveal potential nuclear gene flow between haplogroups I and II.

### Taxonomic interpretation of the discovered pattern

The presence of two sympatric, ecologically differentiated groups within *Hyponephele lycaon* complex in the Middle East is not a completely novel issue. A similar situation is known to exist in Iran and East Turkey (Weiss 1978, Eckweiler and Bozano 2011, Tshikolovets et al. 2014). It is accepted by *Hyponephele* genus experts (Eckweiler and Bozano 2011, Tshikolovets et al. 2014) that in Iran and Turkey these two groups represent two different species: *Hyponephele lycaon* and *Hyponephele lycaonoides* (but see the alternative opinion: Hesselbarth et al. 1995). Although we understand that this point of view requires an additional justification, we may accept it as a working hypothesis until further investigations and taxonomic revisions justify or falsify it.

If the species status of the discovered haplogroups will be confirmed in further studies, we suggest that, following Weiss (1978), the name *Hyponephele lycaon* (Rottenburg, [1775]) can be used for the Israeli taxon characterized by the contrast pattern on the hindwing underside and the predominance of longer brachia in male genitalia. Correspondingly, the name *Hyponephele lycaonoides* D. Weiss, 1978 can be used for the Israeli taxon characterized by the less contrasted pattern of the hindwing underside and the predominance of reduced brachia in male genitalia. However, this nomenclatural decision should be considered as a tentative one. First, despite recent revisions of the genus (Samodurov et al. 1995, 1996, 1997, 1999a, b, 2000, 2001, Eckweiler and Bozano 2011), no one studied type-specimens of numerous taxa that were described as subspecies and variations of *Hyponephele lycaon*. We cannot exclude that the name *lycaonoides* is a synonym of one of the previously described taxa, e.g. of *libanotica* (Staudinger, 1901). Second, molecular markers have never been used for analysis of taxonomic structure of *Hyponephele lycaon* species complex in its whole distribution range. Therefore, we will not be surprised if the true genetic and taxonomic structure of this group will be revealed as much more complex than a simple combination of two sympatric clusters as discovered in Iran, Turkey and Israel.
